# A Novel SERS Substrate Platform: Spatially Stacking Plasmonic Hotspots Films

**DOI:** 10.1186/s11671-019-2928-8

**Published:** 2019-03-13

**Authors:** Li Tang, Yi Liu, Guiqiang Liu, Qiqi Chen, Yuyin Li, Leilei Shi, Zhengqi Liu, Xiaoshan Liu

**Affiliations:** Jiangxi Key Laboratory of Nanomaterials and Sensors, School of Physics, Communication and Electronics, Jiangxi Normal University, Nanchang, 330022 China

**Keywords:** Surface-enhanced Raman scattering, Localized surface plasmonic resonances, Plasmonic hotspots, Raman sensing, Au porous film structures

## Abstract

Surface-enhanced Raman scattering (SERS) technique has presented great potential in medical diagnosis, environment monitoring, and food detection due to its high sensitivity, rapid response, and fingerprint effect. Many efforts have been concentrated on all kinds of strategies to produce efficient SERS platforms. Here, we report a simple and controllable method to produce large-area efficient SERS platforms with spatially stacked plasmonic hotspots. The SERS platforms consist of double-layer metal porous films and are easily fabricated by magnetron sputtering and annealing, assisted by the evaporation of hydrofluoric acid. The stacked dual-layer metal porous films show prominent Raman enhancement and ultrasensitive SERS sensing capability for different target molecules. The detection limit is demonstrated down to 10^−13^ M by detecting rhodamine 6G molecules. These superior Raman properties can be mainly ascribed to the highly dense spatially stacked plasmonic hotspots formed in the dual-layer metal porous films. The simple, controllable, and scalable fabrication strategy and superior Raman performance make these platforms promising candidates for the development of inexpensive, efficient, and mass-produced SERS substrates.

## Background

Surface-enhanced Raman scattering (SERS), as a powerful analytical approach, can provide vibration information of target molecules and has been widely investigated and applied in various fields such as bio-sensing and imaging, medical diagnosis, environmental monitoring, and food detection [1–3]. The SERS technique relies prominently on the localized surface plasmonic resonance (LSPR) of metal nanostructures [4, 5]. It is well known that the free electrons existing in metal nanostructures would oscillate coherently when they interact with the incident photon under certain conditions and thus produce the LSPR [6–10]. The LSPR effects will result in the tremendously enhanced local electromagnetic field near sharp corners/tips and inter-/intra-nanogaps, i.e., plasmonic hotspots [5, 8]. Strongly enhanced electromagnetic field can significantly enhance the Raman signals of target molecules in the hotspots [5, 8], leading to morphology-dependent SERS behaviors and therefore providing the basis for analysis and detection through SERS effects.

To obtain expected SERS platforms with superior Raman performance, various metal nanostructures have been broadly researched by controlling their size, shape, composition, etc. For example, metal nanoparticles, rough metal films, porous nanostructures, periodic arrays, and other hierarchy structures have been fabricated by using electron beam lithography [11], template-assisted technology [12], electroplating [13], and chemical reaction and etching [14]. However, apart from the high cost, sophisticated equipment, and time-consuming synthesis, most of these platforms suffer from a limited density of plasmonic hotspots distributed in either one-dimensional or two-dimensional (2D) space. Increasing the number of plasmonic hotspots is essentially to improve the chances to trap target molecules in the hotspots. Thus, from the practical point of view, one of the key research topics is to develop facile, low-cost methods to construct large-area platforms with high-density plasmonic hotspots.

Three-dimensional (3D) morphological metal nanostructures break the traditional limitation of two-dimensional SERS platforms and supply high-density plasmonic hotspots across all spatial planes within the laser illumination volume [15, 16] and thus increase the versatility of SERS platforms [15]. Nowadays, many strategies have been put forward to build 3D SERS platforms [16]. For example, dielectric nanorods/pillars decorated by Ag nanoparticles have been presented by electroless depositing [17], physical vapor depositing [18], reactive ion etching together with annealing and deposition [4]. These hybrid 3D nanostructures exhibited excellent SERS enhancement due to the formation of high-density plasmonic hotspots with quasi-3D distributed patterns in the metal nanostructures. However, the reproducibility is still an important research direction in the future. The self-assembly of Ag colloidal nanoparticles [15], evaporation of the droplet of citrate-Ag sols on a fluorosilylated silicon wafer [19], and shaking of a mixture of water, decane, and functionalized Ag nanocubes [3] were recently used to obtain 3D SERS platforms. These methods bridge the wet and dry state methods and might allow SERS to be more widely used in various fields. But how to stabilize the constructed 3D geometry of nanoparticles in 3D solutions is still a challenge. Multi-petal flowers assembled by metal nanoparticles as SERS platforms were also engineered by using the spark discharge, ion-induced electrostatic focusing, and e-beam lithography [20]. The number of plasmonic hotspots super-linearly increases with the petal number, and the Raman enhancement is sufficient for the single molecule detection. However, the expensive fabrication device limits its further development. Very recently, porous nanostructures [21, 22] were considered to be high-sensitive SERS platforms. However, the fabrication of these structures needs a long time or complex operation process. Overall, the 3D plasmonic hotspot platforms overcome the long-standing limitations of SERS for the ultrasensitive detection of various target molecules and promise to transform SERS into a practical analytical technique. Therefore, it is greatly vital to design and fabricate optimal plasmonic structures to obtain successful SERS platforms with rich plasmonic hotspots.

In this work, our motif is to design and obtain optimal SERS platforms with spatially stacked plasmonic hotspots. The SERS platforms consisting of double-layer metal porous films are created by integrating simple magnetron sputtering with thermal annealing, assisted by the evaporation of hydrofluoric acid. The SERS dual-layer porous Au films possess excellent fabrication flexibility, scalability and practicability. High sensitivity and good homogeneity are realized in our experiment by detecting different molecules such as rhodamine 6G (R6G), ascorbic acid, and 4-Mercaptobutyramidine (4-MBA). The detection limit is even down to 10^−13^ M for the R6G molecules owing to the appearance of spatially stacked plasmonic hotspots. The experiment results suggest that the dual-layer stacked porous Au films can be used for the novel and practical SERS applications in the biomedicine, food security, and environment detection.

## Methods

### Fabrication

First, ultrathin Au films were deposited on the clean SiO_2_ substrates via the magnetron sputtering. The sputtering time of ultrathin Au films was controlled by the sputtering speed and time. In this work, the sputtering speed was controlled to 32 nm/min and the sputtering time was changed from 19 to 75 s. Then, the sputtered ultrathin Au films together with the clean SiO_2_ substrates were put into the muffle furnace to be annealed at a certain temperature (180–220 °C) for about 30 min with the annealing speed of 2 °C/min. Single-layer porous Au film structures coated on the SiO_2_ substrates were thus formed due to the split and melt of Au material during the annealing process. Next, the SiO_2_ substrate coated with the single-layer porous Au film was placed in a sealed container. A beaker filled with 0.5 M hydrofluoric acid was placed below the samples. By gentle stirring (600 r/min) at room temperature, the hydrofluoric acid vapor would be produced due to the high volatility of hydrofluoric acid. One minute later, the surface of the SiO_2_ substrate was etched and became rough, which greatly reduced the adsorption capacity of Au film. After that, the etched SiO_2_ substrate coated with a single-layer porous Au film was slowly immersed in the de-ionized water until the single-layer porous Au film was separated from the SiO_2_ substrate and fully suspended in the water. Last, another SiO_2_ substrate coated by the same single-layer porous Au film was slowly inserted in the water at a small tilted angle (approximately 45°) to avoid the damage or bending of the suspended single-layer porous Au film during the coating process [23, 24] and then dried at room temperature. Consequently, dual-layer stacked porous Au films were formed on the SiO_2_ substrate. In this work, the fabrication condition and parameters of each layer Au porous film in the dual-layer structures are the same. Through the above steps, the sensitive SERS platforms were prepared by a simple method with no complex procedure involved.

### Structural and Optical Characterization

The morphologies of samples were observed by scanning electron microscopy (SEM, Hitachi, S3400). The samples with double-layer stacked porous Au films were infiltrated into the solution of target molecules with different concentrations for about 24 h and then dried at room temperature. The target molecules included R6G, ascorbic acid, and 4-MBA (all purchased from Aladdin). The Raman spectrometer (Horiba Scientific, LabRRm 750) was employed to measure the Raman signals of target molecules adsorbed in the samples. The spectra were collected using a × 10 objective lens with a numerical aperture of ×100 and an excitation wavelength of 633 nm. The laser powers of 0.061 mW and 0.24 mW were used in this work. The signal collection time was 10 s.

## Results and Discussion

**Scheme 1:**
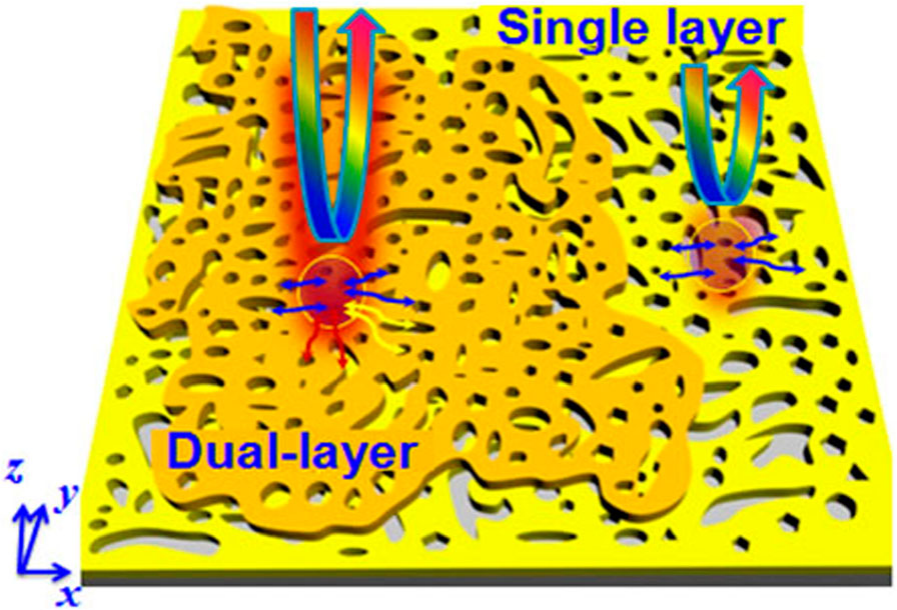
Schematic representation of the dual-layer SERS platform fabricated by stacking another porous Au film (orange) on the pre-prepared single-layer porous Au film (yellow) fabricated under the same conditions and parameters

The proposed dual-layer stacked porous Au film structure with plentiful nanogaps or nanoholes is schematically illustrated in Scheme 1. For identification purposes, two different colors (orange and yellow) were used here to respectively represent the stacked two-layer porous Au films. As we known, the existence of nanoholes or nanogaps in the metal nanostructures provides abundant plasmonic hotspots with extremely enhanced electromagnetic field when they are exposed to the light [21, 22]. On the one hand, the extremely enhanced electromagnetic field contributes to the enhancement of Raman signals of target molecules. On the other hand, rich nanogaps avail the adsorption of target molecules and further enhancement of their Raman signals [4, 5]. For the single-layer porous Au film, quasi-2D distributed plasmonic hotspots in the *xoy* plane is observed. This kind of single-layer porous metal film structures have been investigated widely and presented superior optical property [25]. While for the dual-layer porous Au films, i.e., the single-layer porous Au film coated by another porous Au film with the same parameters, besides the quasi-2D distributed nanogaps occur in the *xoy* plane, ultra-small gaps are also observed in the *z* direction. The 3D distributed plasmonic hotspots are decided by the stacking configuration of porous Au films, which is greatly convenient to the adsorption of many more target molecules in the plasmonic hotspots [26].

**Fig. 1:**
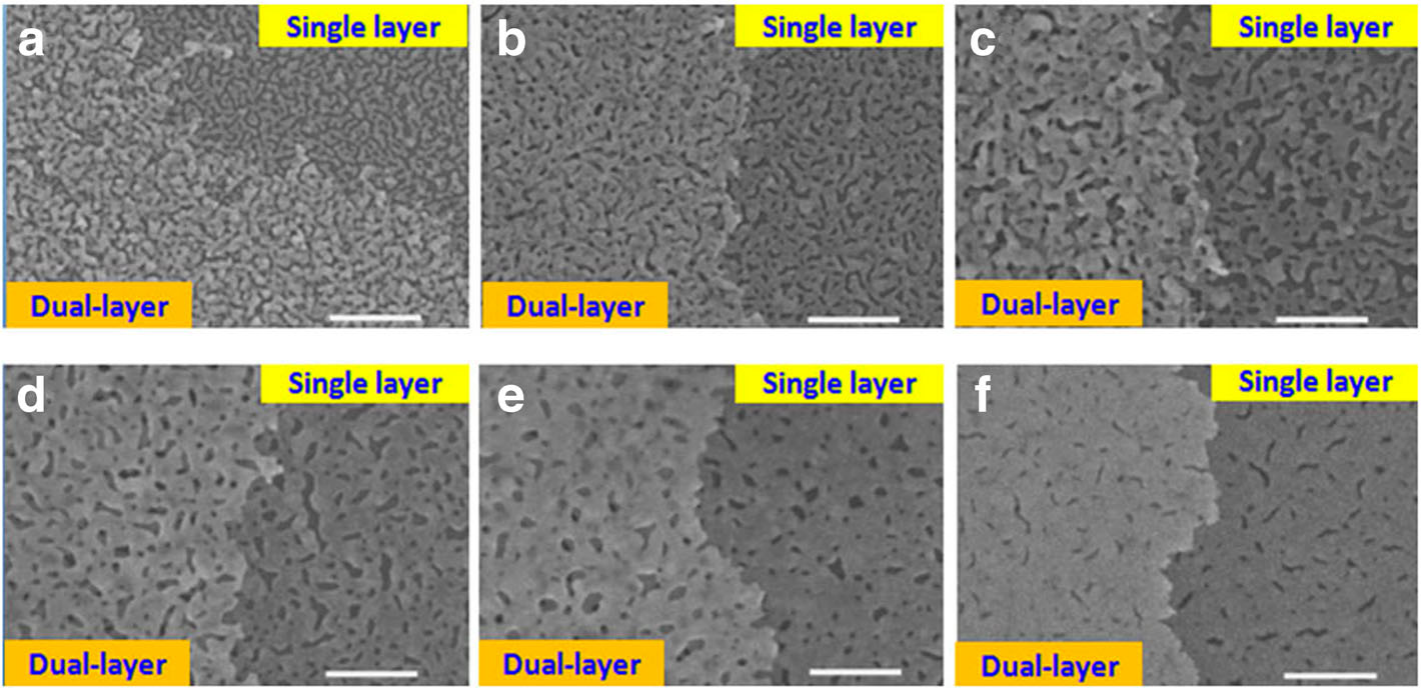
SEM images taken from the boundaries of the fabricated samples where the single- and dual-layer porous Au film structures can be clearly observed. The sputtering time of each layer of porous Au films changes from 19 to 75 s. **a** 19 s, **b** 28 s, **c** 37.5 s, **d** 47 s, **e** 56 s, and **f** 75 s. Annealing temperature: 200 °C. Scale bar is 500 nm

Figure 1a–f shows the SEM images of the fabricated SERS platforms with the sputtering time of each layer of porous Au films increasing from 19 to 75 s. The annealing temperature is 200 °C. For convenient observation, these SEM images were taken from the boundaries of the stacked structures, where the single and dual-layer porous Au film structures can be clearly observed. Ultra-small nanogaps or nanoholes are clearly found in these single-layer and dual-layer porous Au films. Different from the 2D distributed nanohole patterns found in the single-layer domains, prominent 3D distributed patterns of nanoholes are achieved in the dual-layer stacked domains. These SEM images demonstrated the proposed novel SERS platforms with 3D spatially stacked plasmonic hotspots. The formation of single-layer and dual-layer porous film microtopographies can be interpreted by the annealing process to the extremely uniform ultrathin Au films [26], guaranteeing uniform porous Au films which are in favor of the dense and spatially distributed plasmonic hotspots in the 2D and 3D patterns, respectively. The stacked double-layer porous Au films with concentrated 3D plasmonic hotspots are expected to contribute to the optimized and homogeneous SERS signals. When the sputtering time is equal to 19 s, a few Au nanoparticles are found in the sample as shown in Fig. 1a, accompanied by large nanoholes or nanogaps. With the increase of sputtering time, Au nanoparticles disappear and the sizes and number of nanoholes or nanogaps gradually decrease as shown in Fig. 1b–f, which are closely related to the SERS enhancements [27, 28].

**Fig. 2:**
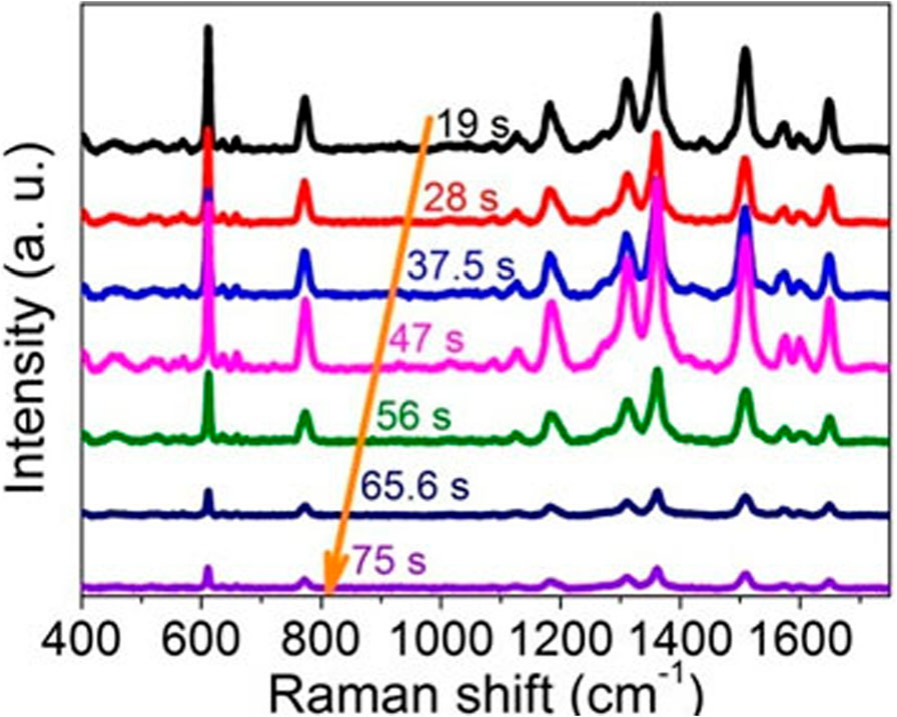
Raman spectra of the dual-layer porous Au film structures with the sputtering time for each layer of porous Au films increasing from 19 to 75 s

Figure 2 shows the Raman performance of dual-layer porous Au film structures by changing the sputtering time from 19 to 75 s. Except where otherwise stated, the laser power in this work is sustained to 0.061 mW, the concentration of R6G molecules is kept to 10^−8^ mol/L, and the annealing temperature is 200 °C. Superior Raman enhancement performance can be observed here when the sputtering time is controlled in the range of 19 to 47 s due to the rich plasmonic hotspots locating at the tips/corners, nanoholes, and edges of dual-layer Au porous films [4–8] as shown in Fig .1. For the sample fabricated by controlling the sputtering time to 19 s, the strong Raman signals can be ascribed to the synergy of LSPRs of Au nanoparticles and nanoholes in this dual-layer structure as shown in Fig. 1a. With the sputtering time increasing from 28 to 47 s, although the Au nanoparticles decrease largely and even disappear, the intensities of Raman peaks gradually increase. It is because that the decreased nanohole sizes contribute to the stronger near-field coupling of LSPRs [4, 5] and thus lead to the enhanced Raman signals, consistent with the results reported in [27, 28]. With the sputtering time increasing to 75 s, the sizes of nanoholes keep decreasing and the number of nanoholes sharply falls, resulting in low-density plasmonic hotspots [3–8, 15–19]. As a result, the Raman signals are largely weakened.

**Fig. 3:**
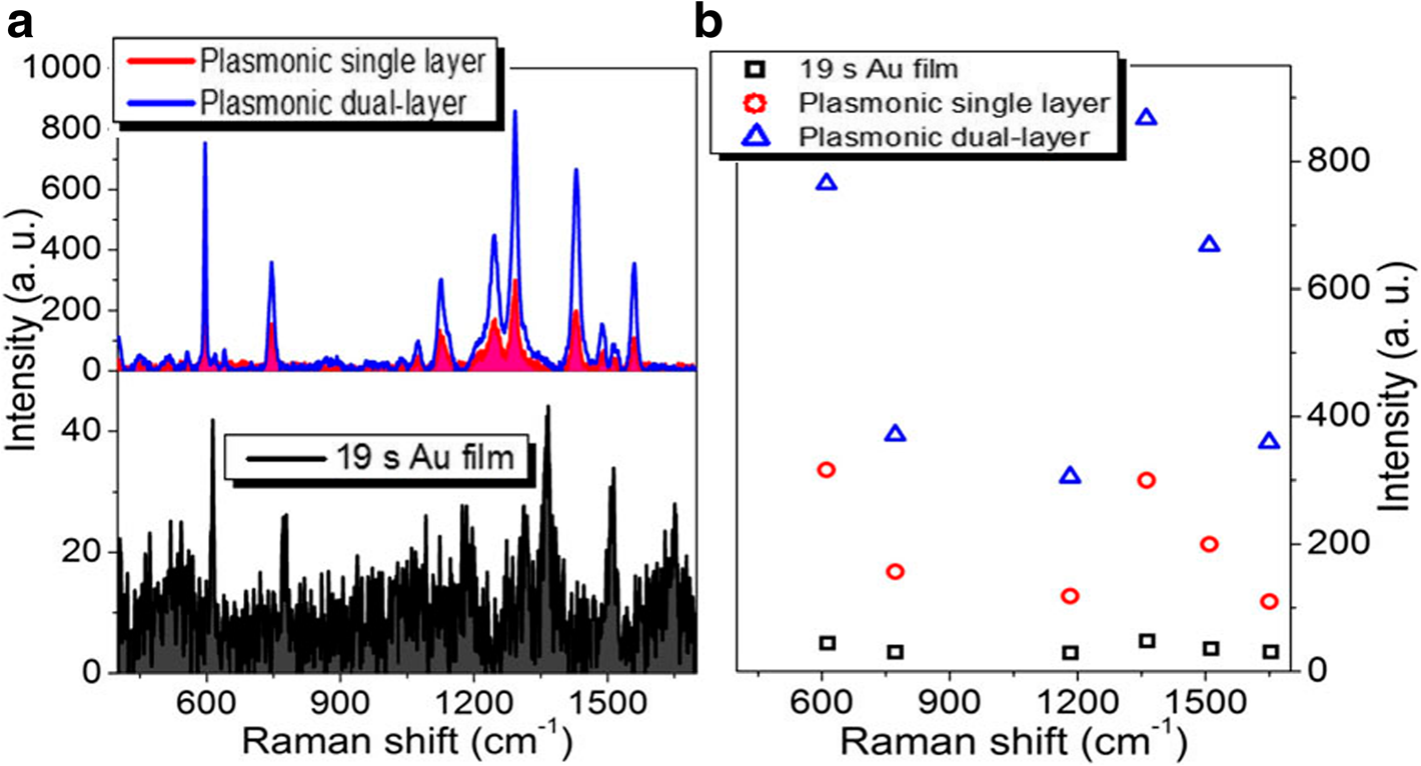
**a** Raman spectra of the single-layer and dual-layer porous Au film structures and ultrathin Au film. **b** Intensity changes of several typical Raman peaks observed in the three Raman curves in **a**. The sputtering time for each layer of porous Au films and film is 19 s

In order to understand the effect of the spatially stacked plasmonic hotspots on the Raman performance, we compare the measured Raman spectra of the single and dual-layer porous Au films with the same sputtering time for each layer of Au film in both structures. Figure 3a shows the Raman properties of R6G molecules adsorbed in the single-layer and dual-layer porous Au films. The sputtering time of each layer of porous Au films is about 19 s. For comparison, the Raman spectrum of R6G molecules on 19 s Au film is also presented here. Obviously, the measured Raman signals of R6G molecules adsorbed in the dual-layer porous Au films are larger than those measured in the single-layer porous Au film and on the ultrathin Au film. Raman intensities of several characteristic peaks (613, 775, 1125, 1368, 1510, and 1653 cm^−1^) in the three Raman curves in Fig. 3a are displayed in Fig. 3b. It is clearly observed that all these characteristic peaks measured in the dual-layer porous Au films are at least 2 times and 15 times larger than those measured in the single-layer porous Au film and Au film, respectively. The largest enhancement of R6G molecules in the dual-layer porous Au films at 1653 cm^−1^ even reaches up to 3.7 times larger than that measured in the single-layer porous Au film. These demonstrate the superior Raman enhancement capability of the dual-layer porous Au films due to the appearance of 3D spatially stacked plasmonic hotspots [4, 26]. That is, the stacked dual-layer porous films with plenty of spatially stacked nanogaps are more conducive for ideal SERS as compared with the single-layer porous Au film reported previously [29].

**Fig. 4:**
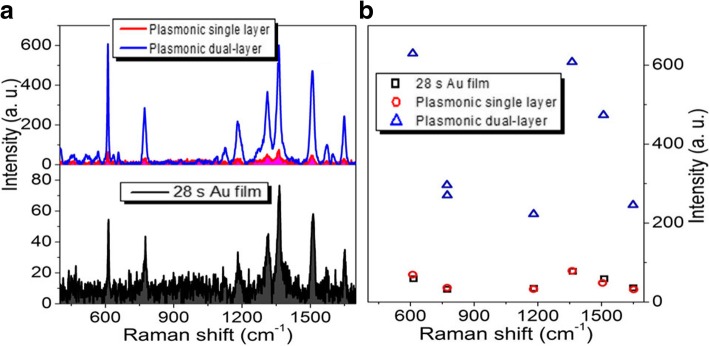
**a** Raman spectra of the single-layer and dual-layer porous Au film structures and ultrathin Au film. **b** Intensity changes of several typical Raman peaks observed in the three Raman curves in **a**. The sputtering time for each layer of porous Au films and film is 28 s

Figure 4a shows the Raman properties of R6G molecules adsorbed in the single-layer and dual-layer porous Au films and Au film. The sputtering time of each layer of porous Au film and Au film is 28 s. Different from those observed in Fig. 3, the Raman signals of R6G molecules adsorbed in the single-layer porous Au film measured here are close to those adsorbed on the Au film. While for the dual-layer porous Au films, the Raman signals of R6G molecules are extremely strengthened. As the Raman intensities of several typical Raman peaks shown in Fig. 4b, the largest and smallest enhancements at 613 cm^−1^ and 1125 cm^−1^ are about 10 times and 5 times larger than those observed in the single-layer porous Au film and Au film, respectively. These again demonstrate the superior Raman enhancement of the dual-layer porous Au films.

**Fig. 5:**
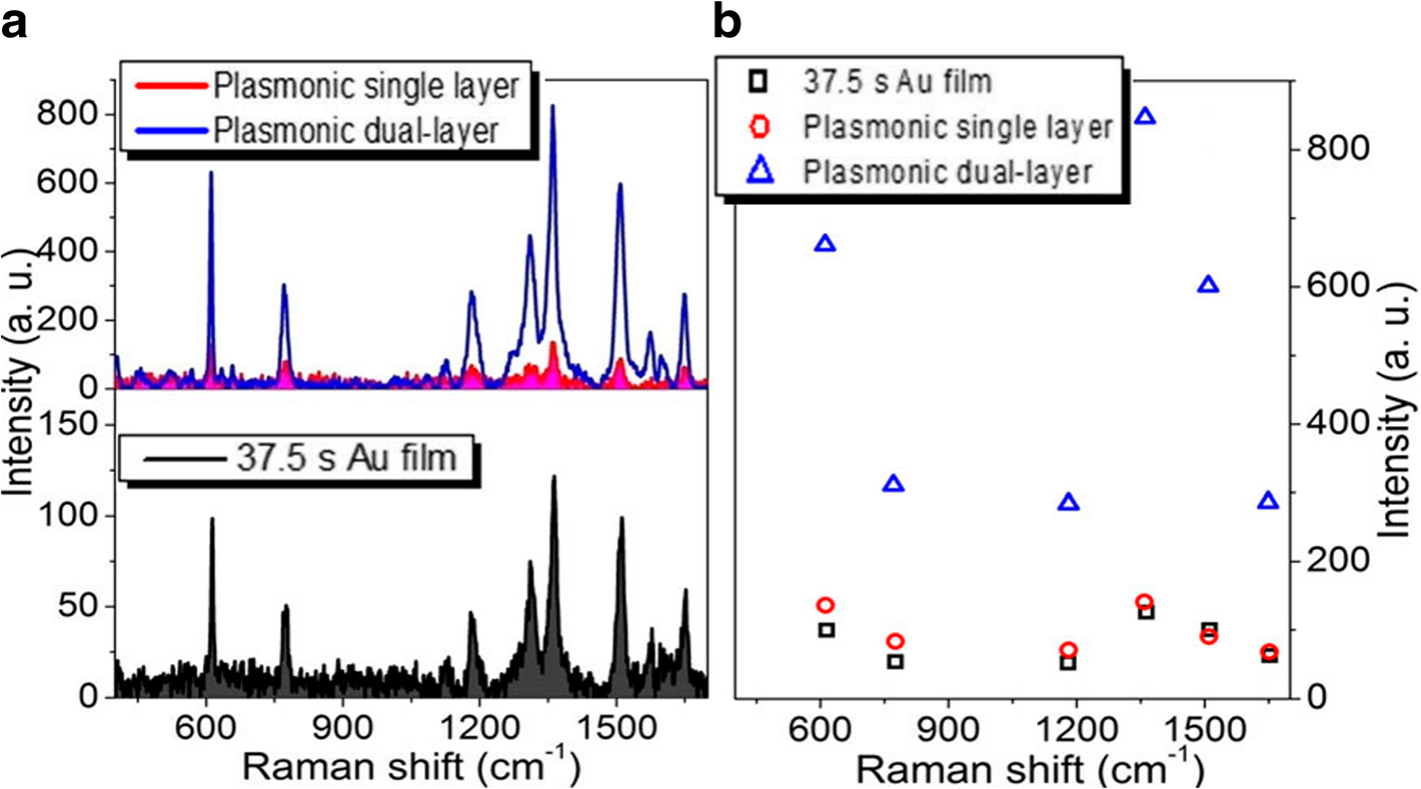
**a** Raman spectra of the single-layer and dual-layer porous Au film structures and ultrathin Au film. **b** Intensity changes of several typical Raman peaks observed in the three Raman curves in **a**. The sputtering time for each layer of porous Au films and film is 37.5 s

Figure 5a shows the Raman properties of R6G molecules adsorbed in the single-layer and dual-layer porous Au films and Au film. The sputtering time of each layer of porous Au film and Au film is 37.5 s. Similar to those observed in Fig. 4, the Raman signals of R6G molecules adsorbed in the single-layer porous Au film are close to those adsorbed on the Au film. While for the dual-layer porous Au films with 3D spatially distributed plasmonic hopspots, the Raman signals of R6G molecules are still extremely enhanced. As the Raman intensities of several typical Raman characteristic peaks shown in Fig. 5b, the largest and smallest enhancements at 613 cm^−1^ and 1125 cm^−1^ are about 7 times and 5 times larger than those observed in the single-layer porous Au film and Au film, respectively.

**Fig. 6:**
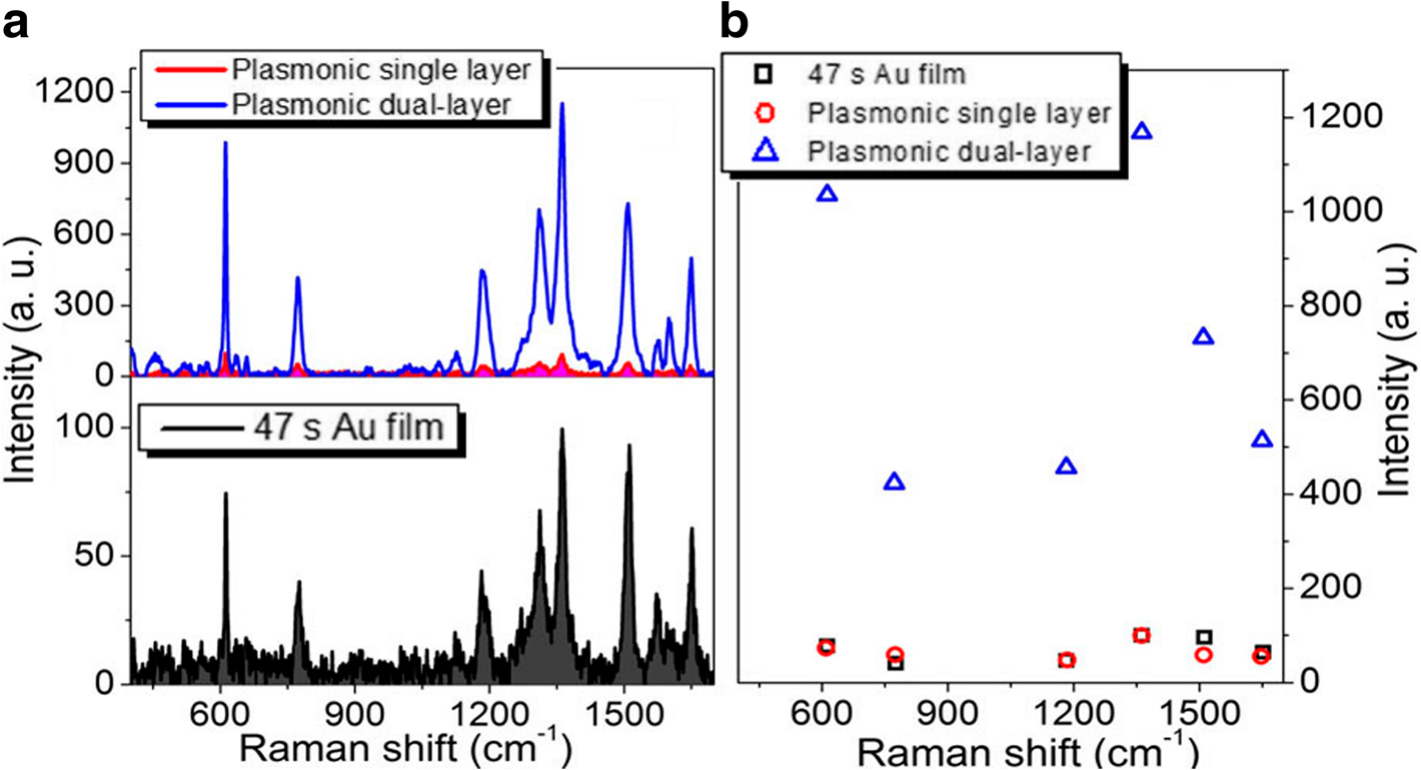
**a** Raman spectra of the single-layer and dual-layer porous Au film structures and ultrathin Au film. **b** Intensity changes of several typical Raman peaks observed in the three Raman curves in **a**. The sputtering time for each layer of porous Au films and film is 47 s

**Fig. 7:**
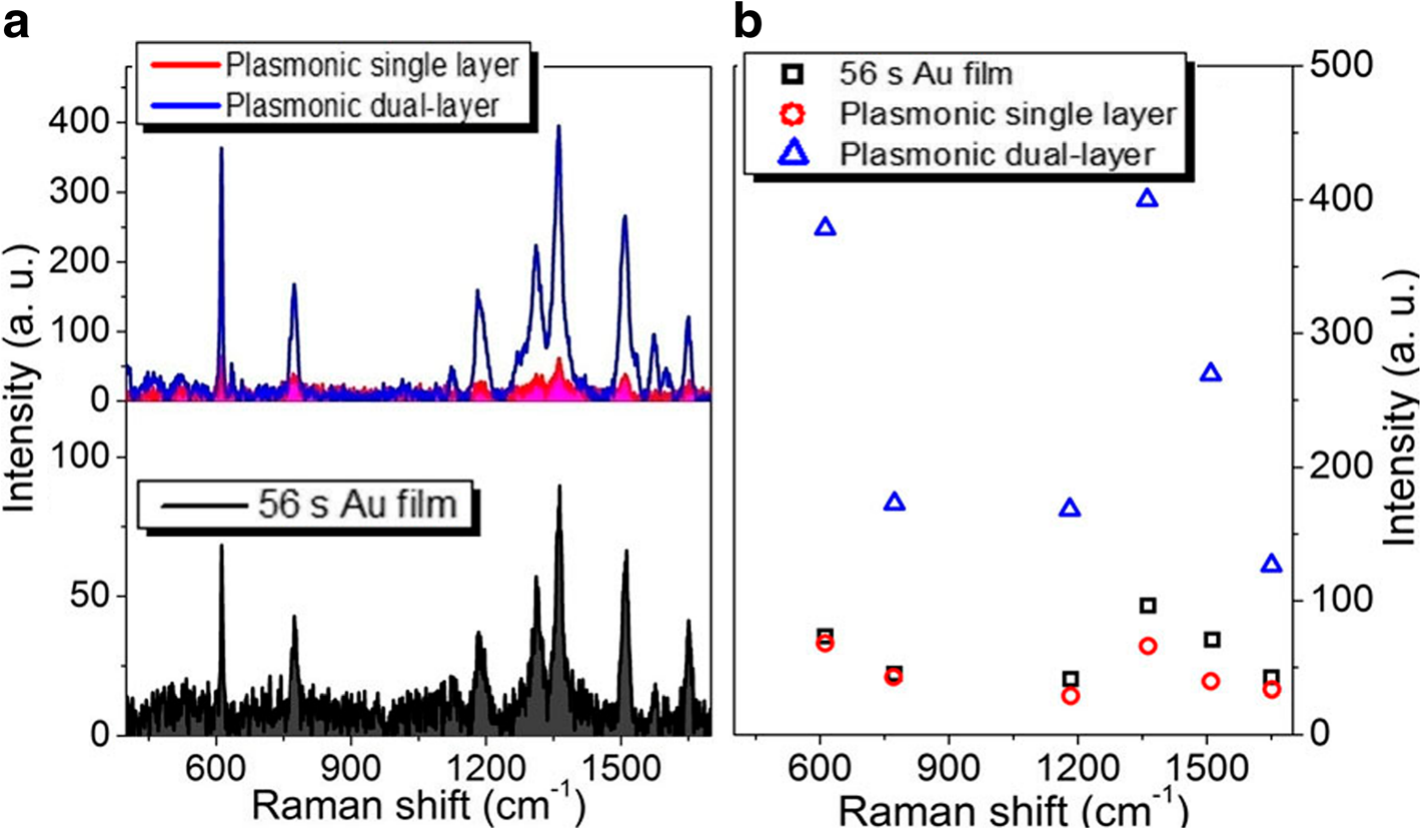
**a** Raman spectra of the single-layer and dual-layer porous Au film structures and ultrathin Au film. **b** Intensity changes of several typical Raman peaks observed in the three Raman curves in **a**. The sputtering time for each layer of porous Au films and film is 56 s

**Fig. 8:**
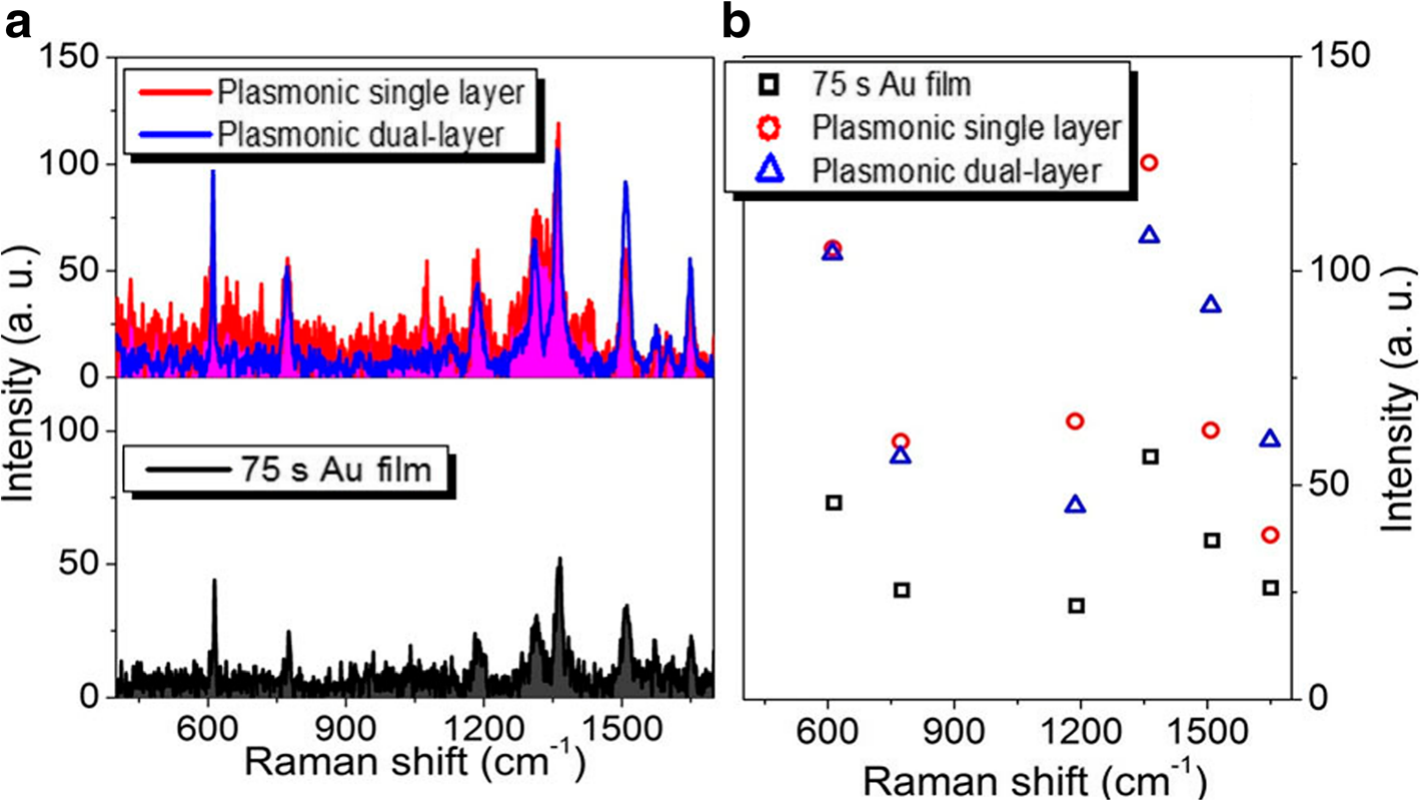
**a** Raman spectra of the single-layer and dual-layer porous Au film structures and ultrathin Au film. **b** Intensity changes of several typical Raman peaks observed in the three Raman curves in **a**. The sputtering time for each layer of porous Au films and film is 75 s

When the sputtering time of each layer of porous Au films and Au film increase to 47 s, the largest and smallest Raman enhancements are about 10 times and 8 times larger than those respectively observed in the single-layer porous Au film and Au film as shown in Fig. 6. With the sputtering time of each layer of porous Au film and Au film increasing to 56 s, although the Raman signals of R6G molecules are still enhanced largely, the largest and smallest enhancements at 613 and 1125 cm^−1^, respectively, become about 4 times and 2 times larger than those observed in the single-layer porous Au film and Au film as shown in Fig. 7. The enhancement effect becomes inconspicuous until the sputtering time of each layer of porous Au film and Au film increases to 75 s as shown in Fig. 8. These indicate the highly scalable fabrication of dual-layer porous Au films when the sputtering time of each layer of porous Au films increases from 19 s to 56 s. The 3D spatially distributed plasmonic hotspots [4, 26] play an important role on the enhanced Raman effects. Noting that, the irregular Raman intensity ratios of characteristic peaks in Figs. 3, 4, 5, 6, and 7 mainly originate from the enhancement inconsistency in the Raman signals between the single-layer and the double-layer porous Au film structures.

**Fig. 9:**
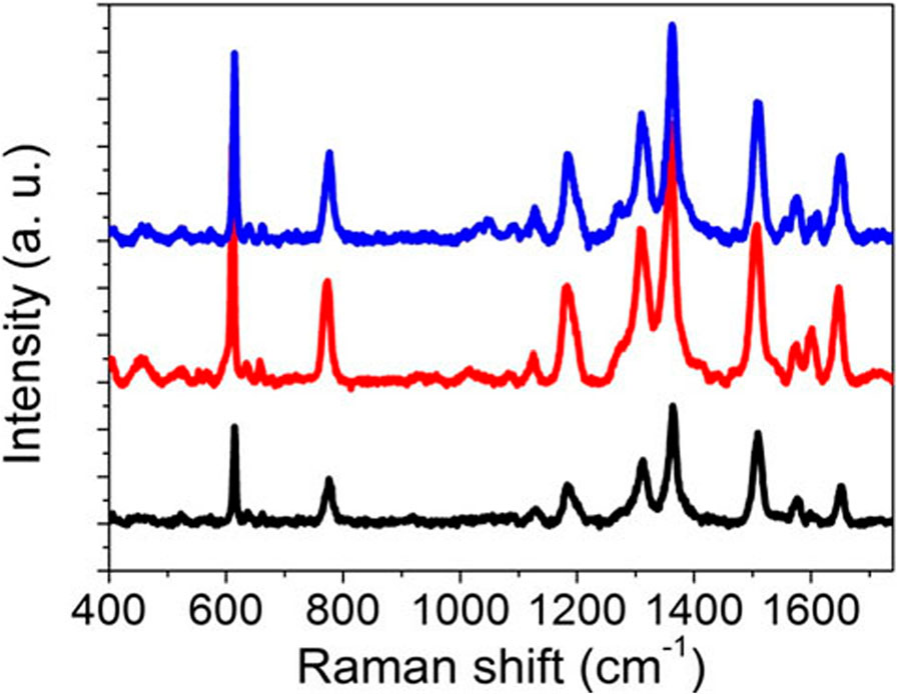
Raman spectra of dual-layer porous Au film structures fabricated at 180, 200, and 220 °C. Laser power: 0.24 mW

The effect of annealing temperature on the Raman enhancement is also investigated as shown in Fig. 9. The annealing temperature changes from 180 °C to 220 °C with a step of 20 °C. The power of 633 nm laser here is 0.24 mW. The sputtering time of each layer of porous Au films is 19 s. It can be easily found that Raman peaks in these spectra are all greatly enhanced. The Raman enhancement effect of the sample fabricated at 200 °C is approximate to that fabricated at 220 °C, which also indicates the scalability of fabricating dual-layer porous Au films at a certain annealing temperature region.

**Fig. 10:**
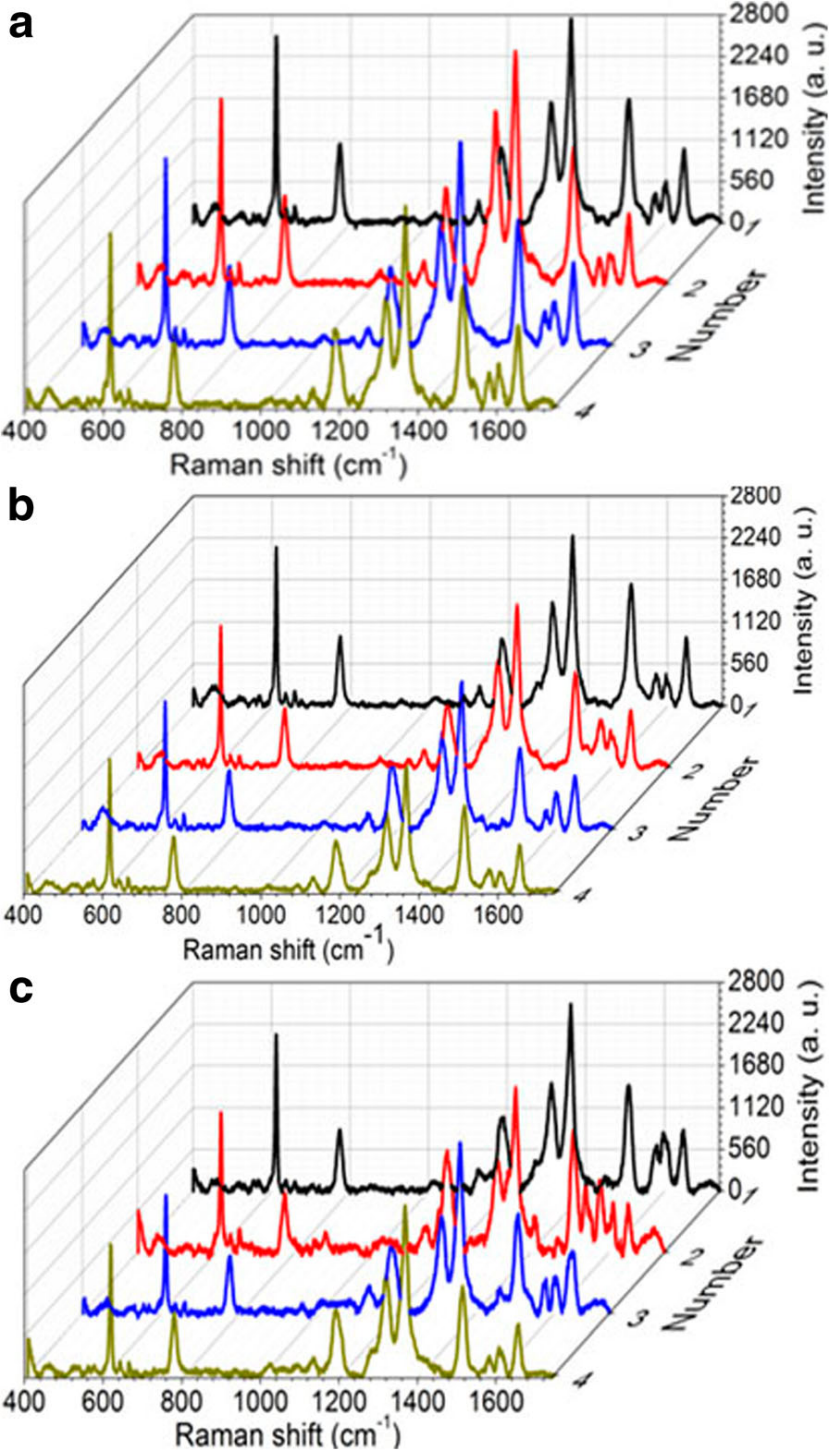
Raman spectra randomly measured at different positions in the dual-layer porous Au film structures with different layer sputtering time. **a** 19 s, **b** 28 s, and **c** 47 s. Laser power: 0.24 mW

The uniformity of the SERS enhancement by this platform was also investigated by randomly selecting four spots in different dual-layer porous Au films for the detection of R6G. The laser power is 0.24 mW. The sputtering time of each layer porous Au film changes from 19 to 28 s and then to 47 s. As we can observe clearly in Fig. 10, there is the same vibration modes of R6G molecules measured from these random points. For example, the uniform peak at 613 cm^−1^ is due to C-C-C ring in-plane bending mode and the peak at 775 cm^−1^ is due to C-H out-of-plane bending mode. Therefore, the decent homogeneity of SERS signals from the proposed dual-layer stacked porous Au films is ascribed to the spatially stacked dense plasmonic hotspots including the hotspots in the horizontally and vertically arranged nanoholes in the porous Au films because of the excitation and coupling of LSPRs. It can thus be concluded that the fabricated dual-layer porous Au films with superior consistency Raman enhancement are conducive to the realization of superior SERS-active platforms.

**Fig. 11:**
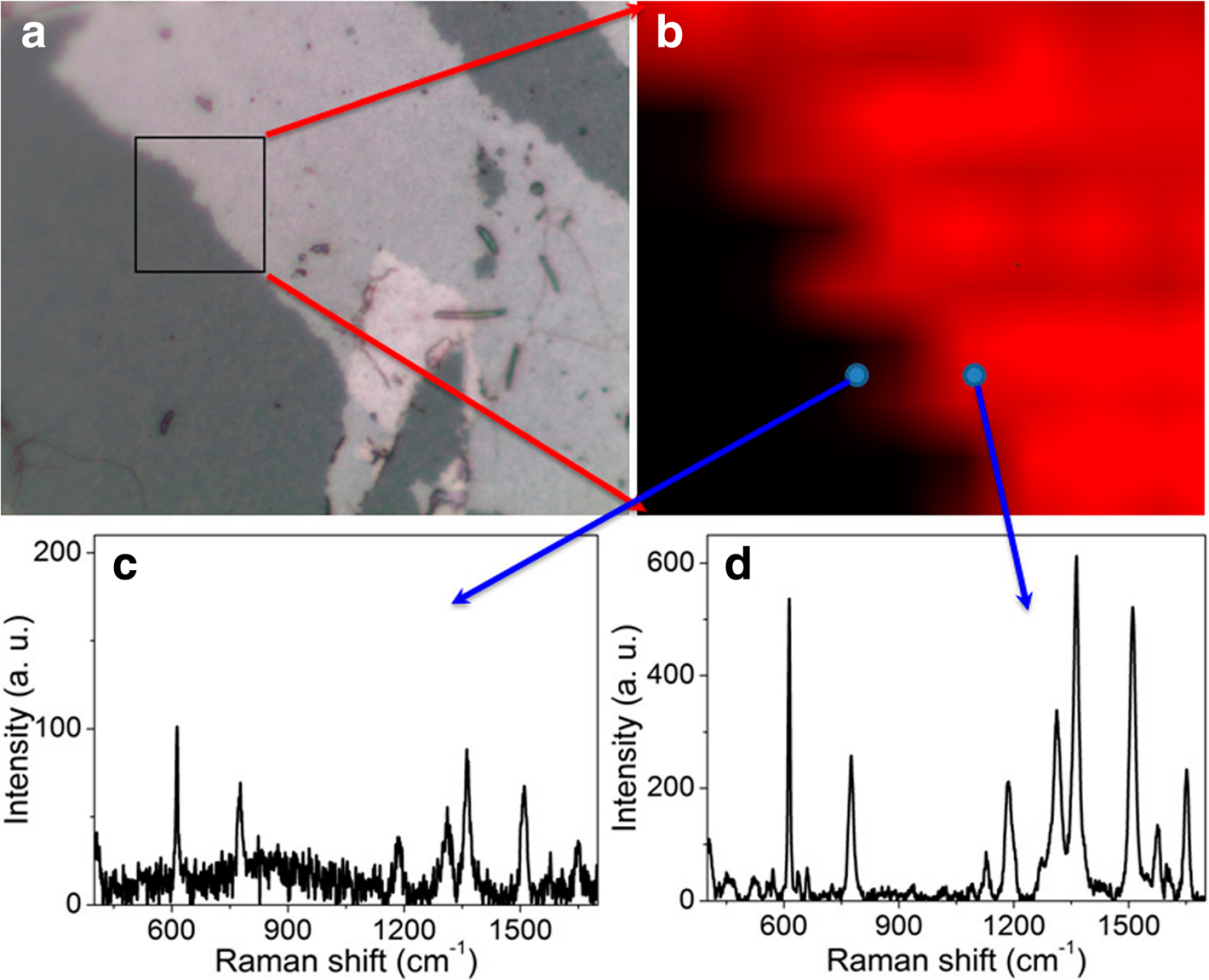
**a** Sample image and **b** SERS mapping taken from the boundary of the fabricated sample with 37.5 s sputtering time of each layer of porous Au films. Raman spectra measured in the single **c** and dual-layer **d** porous Au films as marked with the arrows

Meanwhile, to investigate the influence of the spatial distribution of plasmonic hotspots on the uniformity of Raman enhancement by the dual-layer porous Au films, a point-by-point SERS mapping measurement was conducted on a 15 μm × 15 μm area enclosed by the black square in Fig. 11a for the detection of R6G. The area locates at the boundary of single and dual-layer porous Au films. The left dark domain is the single-layer porous Au film and the right gray domain is the dual-layer porous Au films. The sputtering time of each layer of porous Au films is 37.5 s. The step lengths of mapping in both the *x* and *y* directions were 1.5 μm (10× 10 spots). As the mapping result shown in Fig. 11b, the Raman intensities in the dual-layer porous Au films are much higher than those in the single-layer porous Au film. Moreover, the Raman intensity acquired in the dual-layer porous Au films displays very high uniformity. We can thus conclude that the 3D spatially stacked porous Au films do have a substantial influence on the LSPR efficiency of the porous film structures. Figure 11c and d shows the Raman spectra of R6G molecules measured respectively from the spots in single and dual-layer porous Au film domains as the circles shown in Fig. 11b. As randomly measured places in both single and dual-layer domains in the stacking structures, the stronger Raman signals found in the dual-layer porous Au films confirm the key role of 3D spatially stacked plasmonics hotspots on the uniform Raman enhancement, which is of great significance for potential SERS applications.

**Fig. 12:**
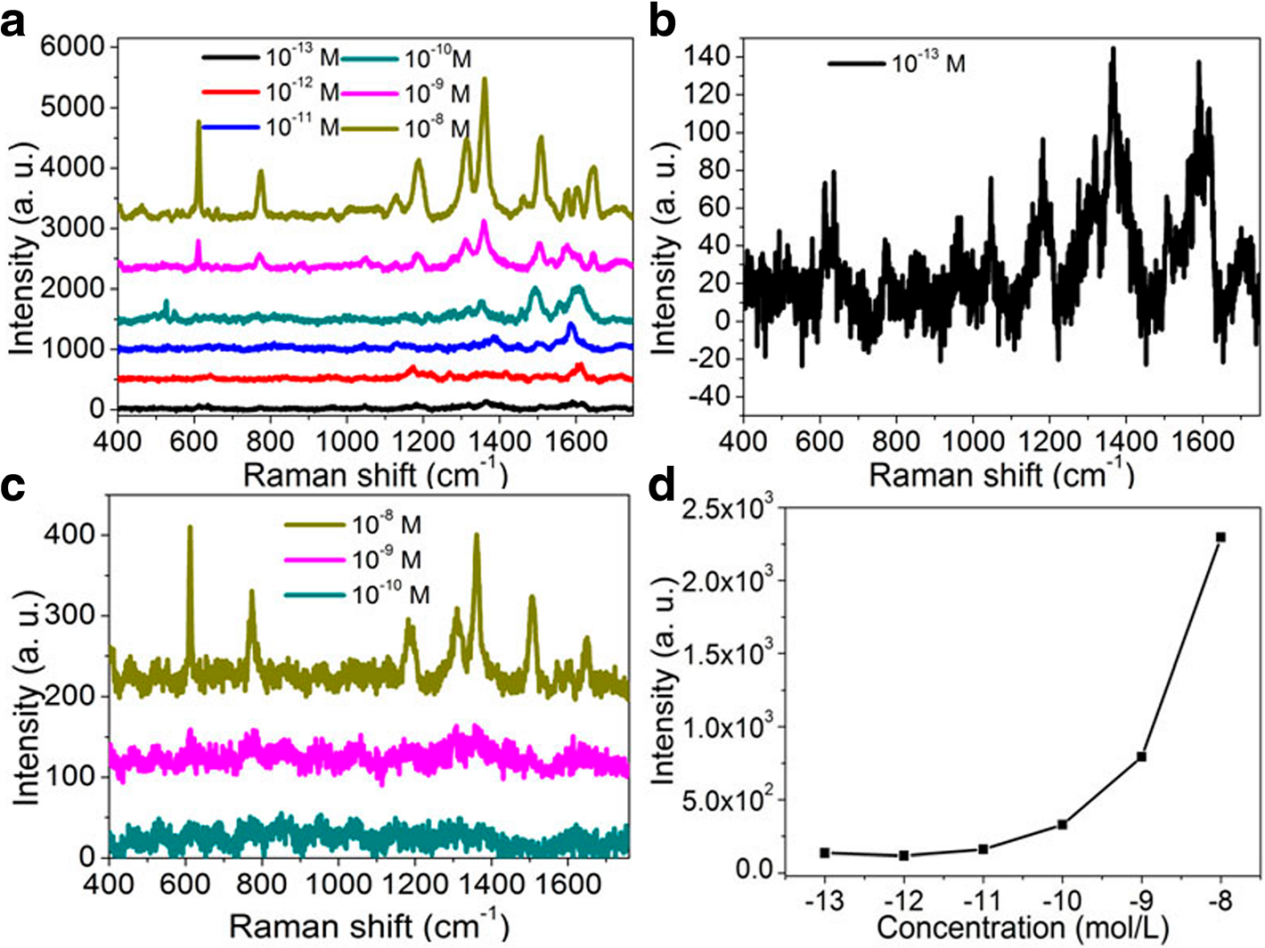
**a** Raman spectra of R6G molecules with the concentration changing from 10^−8^ M to 10^−13^ M in the dual-layer porous Au films. **b** Magnified Raman spectra of R6G molecules with the concentration of 10^−13^ M in the dual-layer porous Au films. **c** Raman spectra of R6G molecules with the concentration changing from 10^−8^ M to 10^−10^ M in the single-layer porous Au film. **d** The relationships of peak intensities at 1368 cm^−1^ and the logarithmic concentrations of R6G. The sputtering time of each layer of porous Au films is 47 s. Laser power: 0.24 mW

The limit of detection of Raman platforms is often confirmed by diluting the concentration of analytes to an extremely low value or even single molecules before the Raman signals completely disappear (i.e., still detectable) [30, 31]. To further evaluate the SERS performance, the detection limit of the fabricated samples was analyzed by changing the concentration of R6G molecules. Figure 12a shows the SERS spectra of the R6G solution with different concentrations in the fabricated dual-layer porous Au films. Here, the sputtering time of each layer of porous Au films is 47 s. The concentration of R6G molecules changes from 10^−8^ M to 10^−13^ M and the laser power is 0.24 mW. As shown in Fig. 12b although some noise exists, the Raman signals can still be distinguished here when the R6G concentration is down to 10^−13^ M, indicating the very high sensitivity of SERS effects and the promising Raman sensing applications by the changes in intensity of Raman peaks. For convenience of observation and comparison, the Raman signals of R6G molecules in the single-layer porous Au films with the concentration changing from 10^−8^ M to 10^−10^ M are presented in Fig. 12c. Different from those obvious Raman peaks observed in the dual-layer porous Au films, for the single-layer porous Au film, the Raman signals of R6G molecules are entirely covered up by the noise signals when the concentration of R6G increases to 10^−10^ M. These again demonstrate the excellent performance of dual-layer stacked porous Au films as compared with the single-layer porous Au film. In addition, the SERS intensity at 1368 cm^−1^ against the logarithmic concentration of R6G is shown in Fig. 12d. An approximate linear relationship of the SERS intensity with the logarithmic concentration of R6G at 1368 cm^−1^ is obtained.

**Fig. 13:**
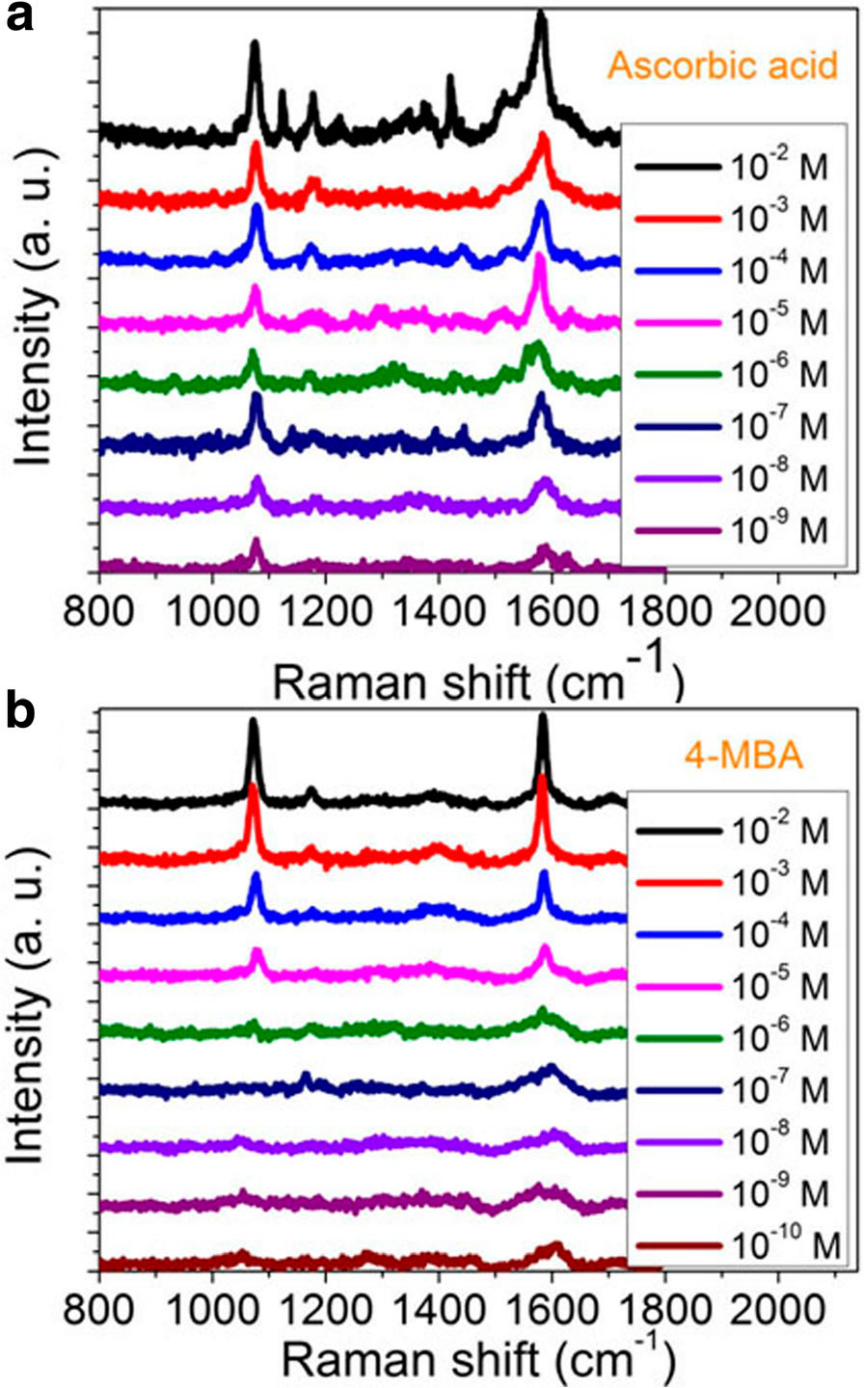
Raman spectra of **a** ascorbic acid molecules with the concentration changing from 10^−2^ to 10^−9^ M and **b** 4-MBA with the concentration changing form 10^−2^ to 10^−10^ M in the dual-layer porous Au films. The sputtering time of each layer of porous Au films is 47 s. Laser power: 0.24 mW

In order to further confirm the SERS sensing capability, we also measured the SERS spectra of ascorbic acid and 4-MBA molecules in the dual-layer porous Au films as shown in Fig. 13a and b, respectively. The concentration of ascorbic acid changes from 10^−2^ to 10^−9^ M and of 4-MBA changes from 10^−2^ to 10^−10^ M. The sputtering time of each layer of porous Au films in these two data is 47 s and the laser power is 0.24 mW. As shown in Fig. 13a, the SERS spectra of ascorbic acid display excellent Raman sensing performance. Two prominent characteristic peaks near 1080 cm^−1^ and 1590 cm^−1^ can be clearly observed even when the concentration of ascorbic acid decreases to 10^−9^ M. These demonstrate the stacked dual-layer porous Au films are also appropriate for the detection of other molecules with low concentration. In Fig. 13b, the Raman peak at 1610 cm^−1^ for the 4-MBA molecules can still be discerned when the concentration is down to 10^−10^ M. These results are comparable to those of many other metal SERS platform, whereas the detection concentration is one or two orders of magnitude lower [32]. These again demonstrate the high Raman sensing performance of the stacked dual-layer porous Au films.

## Conclusions

We have illustrated a facile and economical method for fabricating an excellent mechanical flexible platform based on double-layer-stacked porous Au films as the SERS-active platform. The fabrication of SERS platforms only needs to incorporate the simple sputtering, annealing, stripping, and transfer methods, assisted by the vapor of hydrofluoric acid. High sensitivity and good homogeneity are realized in our experiment by detecting R6G, ascorbic acid, and 4-MBA. The detection limit down to 10^−13^ M is achieved for the R6G molecules adsorbed in the dual-layer porous Au films. Moreover, the SERS dual-layer porous Au films possess excellent fabrication flexibility, scalability, and practicability. The experiment results suggest that the dual-layer stacked porous Au films can be used for the novel and practical SERS applications in the biomedicine, food security, and environment detection.

## Abbreviations

2D: Two-dimension
3D: Three-dimension
4-MBA: 4-Mercaptobutyramidine
LSPR: Localized surface plasmonic resonance
R6G: Rhodamine 6G
SERS: Surface-enhanced Raman scattering
